# Coral rubble facilitates feeding opportunities for invertivorous reef fishes on tropical coral reefs

**DOI:** 10.1007/s00338-026-02909-9

**Published:** 2026-06-30

**Authors:** Lisa S. Goberdhan, Gareth J. Williams, Shaun K. Wilson, Rodney Melanie, Nicholas A. J. Graham

**Affiliations:** 1 School of Ocean Sciences, Bangor University, Menai Bridge, Anglesey LL59 5AB UK; 2 Australian Institute of Marine Science, Crawley, WA Australia; 3 Ocean Institute, University of Western Australia, Crawley, WA Australia; 4 Seychelles Fisheries Authority, Victoria, Mahe Seychelles; 5 Lancaster Environment Centre, Lancaster University, Lancaster, LA1 4YQ UK

**Keywords:** Coral rubble, Reef fish, Foraging ecology, Degraded habitats

## Abstract

Invertivorous reef fishes play a crucial role in coral reef ecosystems, transferring energy from benthic invertebrates to higher trophic levels. They rely on a diverse array of benthic habitats to forage for invertebrate prey. However, climate change and local stressors are reshaping reef habitats, with coral rubble becoming increasingly dominant. Despite low structural complexity, coral rubble supports abundant invertebrates, presenting both opportunities and challenges for invertivorous fish. However, the foraging ecology of fish in rubble habitats remains understudied, limiting our ability to predict their responses to ongoing reef degradation. By combining 28 yrs of reef fish and benthic community monitoring data with targeted rubble disturbance and fish foraging experiments on reefs in the inner Seychelles Islands, we test whether invertivorous reef fish community structure on coral rubble habitats is influenced by reefs characterised by distinct benthic regimes (rubble-dominated, hard substrate-dominated or macroalgal dominated) and by opportunistic feeding mechanisms. We show that invertivorous reef fish communities are highly distinct among benthic regimes at the reef scale, and benthic regime influences the invertivorous reef fish community associated with static, unconsolidated rubble habitats. However, when unconsolidated rubble habitats are physically disturbed, differences among benthic regimes diminish, and a consistent subset of invertivorous fishes rapidly exploit rubble habitats across regimes. These findings are important in shaping our understanding of how reef fish communities may interact with future reef environments, and the species that may have a competitive advantage on rubble-dominated reefs, influencing trophic interactions and resource use.

**Supplementary Information:**

The online version contains supplementary material available at https://doi.org/10.1007/s00338-026-02909-9.

## Introduction

Fishes that feed on benthic invertebrates, broadly referred to as invertivorous fishes, form a distinct and ecologically important group on tropical coral reefs, encompassing some of the most abundant and speciose genera of reef fishes (Williams and Hatcher 1983; Bellwood and Wainwright 2002; Kramer et al. 2015, 2016). Dietary specialisation on invertebrates makes them critical for energy transfer on tropical coral reefs, bridging the gap between benthic invertebrates and higher-level consumers (Kramer et al. 2015, 2016). Invertebrate prey are prolific, comprising the highest density and diversity of metazoans directly associated with coral reef substrates (Plaisance et al. 2009). These substrates encompass all major reef microhabitats including live hard coral, dead coral, coral rubble, turf algae, macroalgae and sand (Enochs and Manzello 2012a; Kramer et al. 2012; Stella et al. 2022). This diverse range of foraging grounds supports a wide variety of invertivorous reef fishes, from small species with highly modified behavioural and morphological adaptations (e.g. the rockmover wrasse and *Novaculichthys taeniourus*) to large-bodied generalist species (e.g. emperor fish, Lethrinidae) (Bellwood and Wainwright 2002; Fulton and Bellwood 2002; Kramer et al. 2015).

In recent decades, coral reefs have experienced unprecedented restructuring due to local human impacts and global climatic stressors (Norström et al. 2016; Hughes et al. 2017; Williams and Graham 2019). Marine heatwaves trigger coral bleaching and disease, resulting in mass mortality of tropical reef-building corals (Hughes et al. 2018; Burke et al. 2023). This has resulted in some reefs transitioning to altered benthic regimes characterised by less diverse coral morphologies, low-lying algal communities, or in some cases, novel configurations like fleshy (non-accreting) macroalgal dominance (McManus and Polsenberg 2004; Graham et al. 2015; Bell et al. 2021; Tebbett et al. 2023). After death, coral skeletons may also rapidly degrade to form coral rubble (Sano et al. 1987; Morais et al. 2022). These rubble habitats are projected to increase in prevalence on coral reefs due to more severe and frequent bleaching-induced coral mortality (Rogers et al. 2014, 2018; Morais et al. 2020; Tebbett et al. 2023). Localised human stressors, such as destructive fishing practices, may also further accelerate the formation of extensive rubble fields (Mcmanus and Jr. 1997, Fox et al. 2003). Altered benthic regimes may impact the foraging ecology of invertivorous reef fish. Similar patterns have been observed in floodplain rivers, kelp forests and grasslands, where a loss of structural complexity reduces refuge from predators and restricts foraging opportunities to a narrower subset of species (Willis et al. ; Janssen et al. 2007,2004; Winkler et al. 2017; Norbury and van Overmeire 2019). Despite being a dominant component of reef fish assemblages, there are limited studies on the foraging ecology of invertivorous fishes and how changes in foraging microhabitats may impact these communities. This is imperative to predicting how invertivorous reef fish communities will respond to ongoing reef degradation and to understanding the broader implications for coral reef food web dynamics, ecosystem stability, and biodiversity conservation.

Coral rubble is formed naturally by wave exposure and bioerosion and its prevalence increases when corals die and become more susceptible to erosional forces. Rubble can accumulate on the reef benthos to form rubble patches interspersed between other reef habitats or large rubble beds (Odum and Odum 1955; Goberdhan et al. 2024). Despite a barren appearance, coral rubble hosts more than an order of magnitude more benthic invertebrates than the same area in live coral (Enochs 2012; Stella et al. 2022). As a result, invertivorous reef fish that can effectively forage in coral rubble will benefit from a disproportionately high supply of prey (Depczynski and Bellwood 2003; Enochs 2012; Enochs and Manzello 2012b; Wolfe et al. 2021). While coral rubble may offer more invertebrate prey than live coral, these habitats are less structurally complex, and therefore do not provide the same predator refuge opportunities as live or even dead but structurally intact coral habitats (Hixon and Beets 1993; Beukers and Jones 1998). Invertivorous reef fishes foraging in coral rubble environments will face a fundamental trade-off between food and safety. Furthermore, prey items in coral rubble are inconspicuously tucked away in the micro-complex architecture of the rubble interstices (Takada et al. 2012; Wolfe et al. 2023a). Therefore, foraging in coral rubble habitats may only be viable for a specific subset of invertivorous fish communities—either those that have specialist morphologies that enable efficient prey capture and handling, or those of larger body sizes, which may mitigate predation risk and facilitate foraging in riskier environments.

Coral rubble can be a physically dynamic habitat, with rubble fragments subject to overturning by hydrodynamic forcing when unconsolidated (Kenyon et al. 2020) or by foraging species that actively disturb the substratum (Sazima et al. 2006, 2007). This dynamism may create feeding opportunities for species who otherwise would not have the morphological and behavioural adaptations to effectively use the habitat for energy acquisition. For example, on sand dunes in the Negev Desert of Israel, winds blow across the dunes, redistributing sand and uncovering seeds. This creates a feeding opportunity for Greater Egyptian sand gerbils (*Gerbillus pyramidum*) to emerge from their burrows and forage for their primary food source. Similarly, army ants (*Labidus praedator* and *Eciton burchellii*), a keystone predator in Neotropical forests, forage in large swarms on forest floors, flushing cryptic arthropods and small vertebrates from leaf litter. In turn, they amass a following of Neotropical birds which use the opportunity to feed on readily available prey (Martínez et al. 2021; Dominguez et al. 2023). Without these opportunistic processes, animals would be required to expend additional energy for food acquisition and increase their vulnerability to predators. As coral rubble cover increases on reefs due to rising coral mortality, and storm intensity increases due to ongoing climate change (Walsh et al. 2016), opportunistic feeding by invertivorous reef fishes may also increase—either due to greater rubble overturning or from fishes capable of overturning rubble themselves. Understanding the reef fish communities that will endeavour to or successfully forage in coral rubble habitats due to opportunistic feeding mechanisms may offer a glimpse into communities that may successfully acclimate and persist on degraded reef conditions.

Despite ongoing coral reef degradation from escalating local human impacts and global climate change, some reefs are predicted to persist, but will do so in altered states, dominated by other benthic forms (Morais et al. 2020; Knowlton 2024), like rubble reefs (Wolfe et al. 2021). The Seychelles inner Islands reefs is a prime example of this. Reef fish and benthic communities have been monitored over 28 yrs, revealing long-term dynamics in reef conditions, ranging from changes in dominance amongst live coral, coral rubble and macroalgae (Chong-Seng et al. 2014; Wilson et al. 2019; Graham et al. 2024). By combining this long-term dataset with targeted rubble disturbance and fish foraging experiments, our overarching aims were to test (1) whether invertivorous reef fish community structure (species, body size, and trophic guild) differs across benthic regimes, (2) if the community structure of invertivorous reef fish present on and foraging in coral rubble habitats is influenced by the physical disturbance of rubble, and (3) whether the community structure of invertivorous reef fish present on and foraging in coral rubble habitats is influenced by their corresponding benthic reef regime. By doing so, we provide key insight into invertivorous reef fish communities associated with degraded coral habitats, and novel insights into their foraging ecology. Our findings indicate that while static coral rubble habitats offer limited foraging opportunities for invertivorous reef fishes, these constraints are significantly reduced when unconsolidated rubble is disturbed, expanding foraging opportunities across a broader range of species and body sizes.

## Materials and methods

### Quantifying coral reef benthic and reef fish communities in Seychelles (1994–2022)

The Seychelles consists of a mix of granitic and carbonate islands in the western Indian Ocean. The inner islands (4.5° S, 55.5° E) rise from the Mahe Plateau (20–70 m depth) and are predominantly granitic with well-developed carbonate fringing reefs (Braithwate 1971). Benthic and fish community composition were monitored at 18 reefs in the inner Seychelles Islands in each of 7 yrs (1994, 2005, 2008, 2011, 2014, 2017 and 2022). These reefs have a history of disturbance with a marine heatwave in 1998 resulting in mass coral bleaching and 90% loss of live coral (Graham et al. 2006b). Some of these reefs underwent regime shifts from hard coral dominated to macroalgal-dominated states, or became rubble-dominated with low cover of living macrobenthos, while others recovered to relatively high live coral cover (Chong-Seng et al. 2014; Graham et al. 2015). Another marine heatwave in 2016 also caused a mass coral bleaching event, and a 70% decline in coral cover (Wilson et al. 2019). By 2022, most macroalgal-dominated reefs remained in their altered stable states, one regime-shifted reef recovered back to live coral-dominated state and the remainder are recovering to live coral cover (Graham et al. 2024).

At each site, eight to sixteen replicate 7-m radius point counts were conducted to survey fish and benthic communities along the reef slope at 2-9 m depth. Within each point count area, the percent cover of benthic categories including hard coral, macroalgae, coral rubble, sand, dead coral, soft coral and rock, was quantified in situ based on a diver’s planar view of the area. In addition, structural complexity of the reef within each area was ranked between 0 and 5, where 0 represents rubble or sand reefs with little complexity and 5 highly complex reef structures with caves and overhangs (Polunin and Roberts 1993). This visual approach has been validated against other common methods of estimating structural complexity and produces comparable results (Wilson et al. 2007). The density and individual sizes (estimated as length) of diurnally active, non-cryptic, reef associated fish were also recorded within each point count area. Length estimation was calibrated at the beginning of each survey day with the same diver visually estimating the length of known lengths of PVC pipe. Accuracy was within 4% of actual lengths. All surveys were conducted by three experienced scientists (Simon Jennings, SKW, NAJG).

### Quantifying invertivorous reef fish present and feeding on coral rubble habitats (2022)

**Fig. 1:**
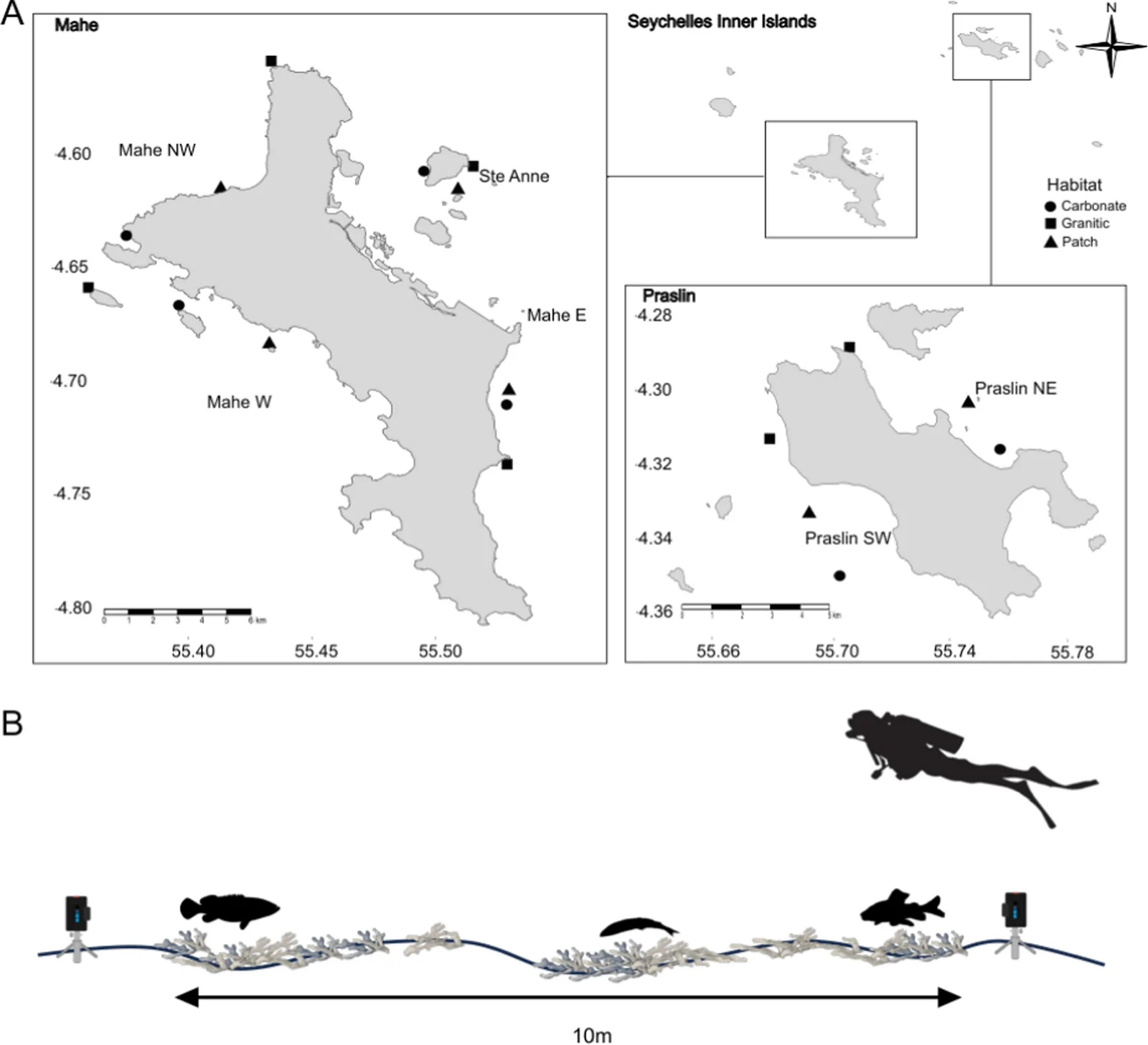
**a** Long-term survey sites (*n* = 18) around the inner Seychelles Islands of Mahe, Ste Anne and Praslin. **b** Video cameras placed at each end of a 10 m transect (*n* = 1–3 transects per site) to capture invertivorous reef fishes present or feeding in coral rubble habitats

Rubble disturbance experiments were conducted at the same sites used to characterise long-term changes in fish and benthic communities in the inner Seychelles Islands in March 2022, with the exception of one site (Mahe NW Granitic), where experiments were aborted due to a logistical issue. Consequently, experimental survey data were collected from 17 sites. Rubble habitats were located haphazardly at each site and deemed suitable if there was at least a 10 m stretch containing patches of rubble (where a rubble patch was at least 0.5 m × 0.5 m). Hereafter, ‘rubble habitats’ refers specifically to rubble patches within the experimental transects. When a suitable area was found, a 10-m transect tape was laid on the substrate. A GoPro^©^ Hero 4 mounted on a tripod (reaching an approximate height of 15 cm above substrate) was placed at each end of the transect, pointing directly down the centre of the transect line, and recorded video continuously (Fig. 1b). Divers then swam approximately 5 m away from the video setup to avoid disturbing fish behaviour. After 3 min, a diver manually disturbed the rubble habitat substrate along the entire transect line using a plastic scoop to quickly scoop and overturn rubble while traversing the transect line. This manual disturbance was intended as a standardised disturbance assay reflecting the physical dynamism of coral rubble and its susceptibility to overturning under natural processes such as wave action (Kenyon et al. 2020) or the feeding activity of larger animals (Sazima et al. 2006, 2007). While the experiment does not replicate any single natural process, it provides a controlled and repeatable proxy to assess which invertivorous fish species are willing to approach and feed in recently disturbed rubble habitats. After manual disturbance was completed along the transect line, the diver swam away again. The camera was left on for a further 3 min, before being turned off. The 3-min time constraint was imposed by logistical limitations on available dive time and was applied consistently across transects to ensure standardised comparisons among sites. This was repeated twice at different rubble habitats within each site, for a total of three transects per site.

For each transect, video recordings from both cameras were reviewed separately in VLC media player at a reduced playback speed to identify and enumerate invertivorous reef fishes. At most sites, video visibility exceeded 5 m. Therefore, for each camera, fishes were counted within an approximate 5-m-long field of view, corresponding to half the length of the 10-m transect line. Fish presence was recorded along the entire transect line, which included both rubble and non-rubble habitats. Individual fishes frequently moved between rubble habitats and adjacent non-rubble habitats along the transect line during the observation period. Recording fish presence along the full transect therefore captured which species were present on and around surrounding rubble habitats. The number of fishes observed within the video were counted per species pre-rubble disturbance (initial 3 min of video footage after divers swam away) and post-rubble disturbance (latter 3 min of video after rubble was manually disturbed). If an individual fish exited the frame and then immediately re-entered the frame, it was not counted again. For each counted fish, a behavioural observation was recorded as either ‘feeding’ (defined as swimming towards the rubble habitat, angling its head downward, and actively biting the rubble) or ‘not feeding,’ both pre- and post-disturbance. Only feeding events that occurred on rubble habitats were recorded and analysed. The total number of fish from each camera was then tallied to generate an overall fish count by species for each transect. Fish body size was applied based on mean species-level body lengths from long-term reef fish survey data (1994–2022), as no accurate size reference was available in the video footage.

### Statistical analyses

#### Identifying benthic regimes on coral reefs (1994–2022)

To simultaneously visualise correlation across all benthic variables, we performed a Principal Component Analysis (PCA) on the benthic community data. Structural complexity was also included as a benthic variable as it is a key indicator of reef habitat quality (Graham and Nash 2013). Data from each point count were averaged across site and year combinations (here-on referred to as site-year combination), such that each site–year combination represents a single observational unit reflecting the benthic state of a reef in a given year. Data were standardised to account for variables measured at different scales. Statistical grouping of the benthic variables was conducted using a K-means partitional clustering process using the *k-means* function in the base R stats package in R programming language version 4.2.3 (R Core Team 2023). This is an unsupervised machine learning algorithm used for partitioning a dataset into a pre-defined number of clusters (*k*). The algorithm is centroid-based and aims to minimise the sum of distances (Euclidean in this study) between the points and their respective cluster centroid (Sinaga and Yang 2020). Prior to conducting the clustering method, we used the Elbow Method to determine the optimal number of pre-defined clusters (*k*). This method calculates the sum of the squared distances between datapoints and their cluster centroid (termed within cluster sums of squares—WCSS), with increasing numbers of *k*. The data are graphically represented, and the inflection point (or ‘elbow’) is taken as the optimal number of clusters—this is the point at which increasing *k* leads to only marginal reduction in WCSS (Xu et al. 2016). Benthic regimes were then assigned based on mean values of benthic cover in each cluster.

#### Testing for differences in invertivorous fish community structure across benthic regimes (1994–2022)

We tested for and visualised differences in invertivorous fish community composition across the benthic regimes (identified using the *k-means* clustering function) using a constrained canonical analysis of principal coordinates (CAP) (Anderson and Willis 2003). CAP was appropriate because it maximises group separation and objectively measures community distinctness by finding axes through the multivariate cloud of points that best discriminate among a priori defined groups (benthic regimes in this case). It uses a leave-one-out procedure to estimate the allocation success of the CAP model placing data points in the correct group (Anderson et al. 2008). Allocation was considered more indicative of a distinct community type than expected by chance alone when values exceeded 33.3%. This threshold came from the possibility of each individual observation having a 33.3% chance of being randomly placed back into its original benthic regime. CAP analyses were based on 9999 permutations of the raw data. Individual species driving group differences in the CAP plot were investigated by calculating Spearman Rank correlations of the canonical ordination axes with the original variables (fish species). Species with correlations (≥ 0.2) were then overlaid as a biplot.

We then tested for an effect of benthic regime (fixed factor, 3 levels) on invertivorous fish body size (univariate response) and on each invertivorous fish trophic guild abundance (univariate responses) using a permutational analysis of variance (PERMANOVA) (Anderson 2001) using the PERMANOVA + add-on for PRIMER (version 7.0) (Anderson et al. 2008). Analyses were based on a Euclidean similarity matrix and 9999 permutations of the residuals under a reduced model. Pairwise PERMANOVA tests were used to explore significant differences between factor levels. We used PERMANOVA instead of CAP because CAP is designed for ordination and dimensionality reduction, requiring multiple dimensions to produce meaningful axes. In contrast, PERMANOVA can be applied to univariate response variables, where it is mathematically equivalent to a one-way ANOVA, making it the appropriate choice for our univariate analyses. Species-specific body size estimates were calculated as the mean length of each species from the surveys conducted from 1994 to 2022. Invertivorous trophic guilds were also assigned to each species following Parravicini et al (2020), a study that compiled the gut content of reef fish globally (Table S1). These trophic guilds include crustacivores—fish species that primarily feed on large Crustacea (Decapoda and Stomatopoda), macroinvertivores—fish species that primarily feed on Mollusca (Bivalvia, Gastropoda, Polyplacophora, and Scaphopoda), Echinoidea, and Ophiuroidea, microinvertivores—fish species that primarily feed on Arachnida, Pycnogonida, small Crustacea (Peracarida), and worms (Annelida, Hemichordata, Nematoda, Nemertea, and Sipuncula), sessile invertivores—fish species that predominantly feed on sessile invertebrates (e.g. Asteroidea, Bryozoa, and Porifera) (Parravicini et al. 2020).

#### Testing the effect of rubble disturbance and benthic regime on invertivorous fish community structure on coral rubble habitats (2022)

We re-ran the K-means clustering analysis to assign benthic regimes for sites only in the year 2022. We also re-ran a CAP analysis to determine the distinctness of invertivorous fish communities across the benthic regimes in 2022. This was necessary to ensure that benthic community data and fish community composition reflected the current conditions and did not contain confounding effects of temporal regime shifts and other dynamic trajectories which occurred in Seychelles following mass coral bleaching events (Graham et al. 2015; Wilson et al. 2019). Individual species driving group differences in the CAP plot were investigated by calculating Spearman Rank correlations of the canonical ordination axes with the original variables. Based on its correlation coefficient and position along the CAP axes, each species was assigned a ‘regime-association’. The regime-association was useful in visualising how many species associated with a specific regime showed up in other regimes during the rubble habitat experimental surveys (for example how many macroalgal-associated invertivorous fish species were present on rubble habitats at hard substrate benthic regimes).

We tested for an effect of rubble disturbance (fixed factor, 2 levels) and benthic regime (fixed factor, 3 levels; with site nested) on invertivorous fish community composition using PERMANOVA. Analyses were based on untransformed data (since raw data only ranged from 0 to 2 for each replicate), a zero-adjusted Bray Curtis similarity matrix and 9999 permutations of the residuals under a reduced model. The zero-adjusted Bray Curtis was used as it is recommended when two sample units are empty (i.e. equal to zero) (Clarke et al. 2006). In this case, we would expect two sample units to be empty if there was no effect of disturbance on the community. The zero-adjusted Bray Curtis measure adds a dummy species to every sample unit prior to calculation of the dissimilarity matrix and calculates two empty sample units as 100% similar (Clarke et al. 2006). After running this initial analysis, we decided to make a couple of adjustments to improve the design.

Firstly, at the individual replicate scale (transect), the data in the resemblance matrix were extremely uninformative since many contained zero fish, therefore many cells in the zero-adjusted Bray Curtis resemblance matrix equalled 100% similarity. We rectified this by averaging the data across the transects for each site which considerably improved the information content in the resemblance matrix by reducing the number of cells with 100% similarity. Therefore, we decided to run all analyses at the site level rather than replicate level. Secondly, we expected a significant effect for the interaction between disturbance and benthic regime, as the raw data showed a clear difference in pre-disturbance community composition across benthic regimes (Fig. 4a). However, low replication at the benthic regime level coupled with relatively high variability among the individual sites precluded the detection of any regime effect. Since the interaction effect of disturbance and site (nested in regime) was significant, we were justified in examining benthic regime (fixed factor, 3 levels) and site (nested in benthic regime; 17 levels) separately within each disturbance level (i.e. a separate analysis for pre-disturbance and post-disturbance data). As such we redesigned our analysis as follows. We first tested for an effect of rubble disturbance (fixed factor, 2 levels) on invertivorous fish community composition, then we tested for an effect of benthic regime (fixed factor, 3 levels) and site (random factor nested in benthic regime, 17 levels) on pre-disturbance and post-disturbance data, respectively.

The same analyses were run on univariate community data (invertivorous fish mean body size, invertivorous fish standard deviation body size and abundance of each trophic guild). Analyses were based on untransformed data, a Euclidean similarity matrix and 9999 permutations of the residuals under a reduced model. We then repeated the same analyses for invertivorous fishes feeding in rubble habitats (community composition, mean body size, standard deviation body size and abundance of each trophic guild). Three sites contained only one transect each due to technical issues with the video cameras. The model was first run with all sites included, and then re-run with these three sites removed as a form of sensitivity analysis to determine if the unbalanced experimental design affected the overall results. The results of our sensitivity analysis gave the same effects; and therefore, the imbalance in the experimental design did not affect our test results. Pairwise PERMANOVA tests were used to explore significant differences between factor levels, and similarity percentage analysis (SIMPER) using Bray Curtis distance was used to determine invertivorous fish species that contributed most to between-group dissimilarities (Anderson et al. 2008). SIMPER analysis was used instead of CAP because SIMPER identifies the species that contribute most to the dissimilarity between groups, whereas CAP, as an ordination technique, focuses on visualising group separation but does not quantify species’ contributions to dissimilarity.

## Results

### Benthic regimes on Seychelles’ coral reefs (1994–2022)

**Fig. 2:**
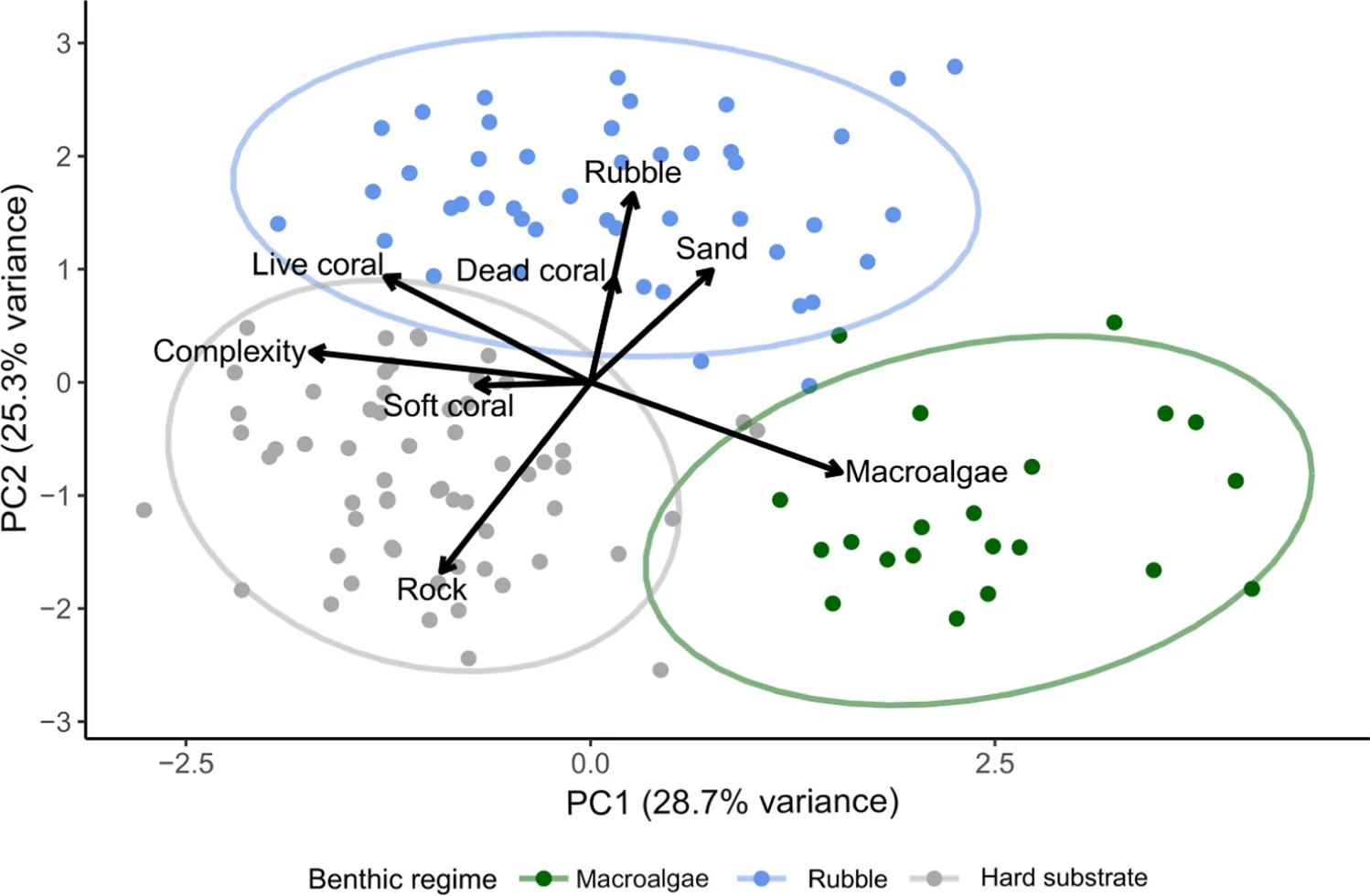
Principal components analysis (PCA) with the K-means clustering of points overlaid. Each point represents a site-year combination, i.e. benthic community data averaged over 8–16 point count surveys at a site in a year (1994–2022). Green dots (macroalgal regime, *n* = 21), blue dots (rubble regime, *n* = 45), grey dots (hard substrate regime, *n* = 60) (Table 1). The ellipses encompass 80% of the dots associated with each benthic regime

There were strong correlations between hard coral and macroalgal cover, and these two variables negatively correlated along PC1, which explained 28.7% of the variation in the data (Fig. 2). Rubble and rock were also negatively correlated along PC2, which accounted for 25.3% of the variation in the data (Fig. 2). There were also positive correlations among the benthic variables, with rubble strongly correlated with sand, and hard coral strongly correlated with structural complexity (Fig. 2).

We identified three major benthic clusters in the benthic data (Fig. 2, Fig. S1). The first cluster consisted of 21 site-year combinations dominated by macroalgae (48.8%), the second cluster consisted of 45 site-year combinations dominated by rubble (36.0%) and much higher hard coral cover (19.3%) than the first cluster, and the third cluster consisted of 60 site-year combinations dominated by rock (58.7%) and similar hard coral cover (15.2%) to the second cluster (Fig. 2, Table 1). Based on the dominant benthic habitats in each cluster, the following three benthic regimes were assigned as: (1) macroalgal regime, (2) rubble regime, and (3) hard substrate regime (Fig. 2, Table 1).

**Table 1:**
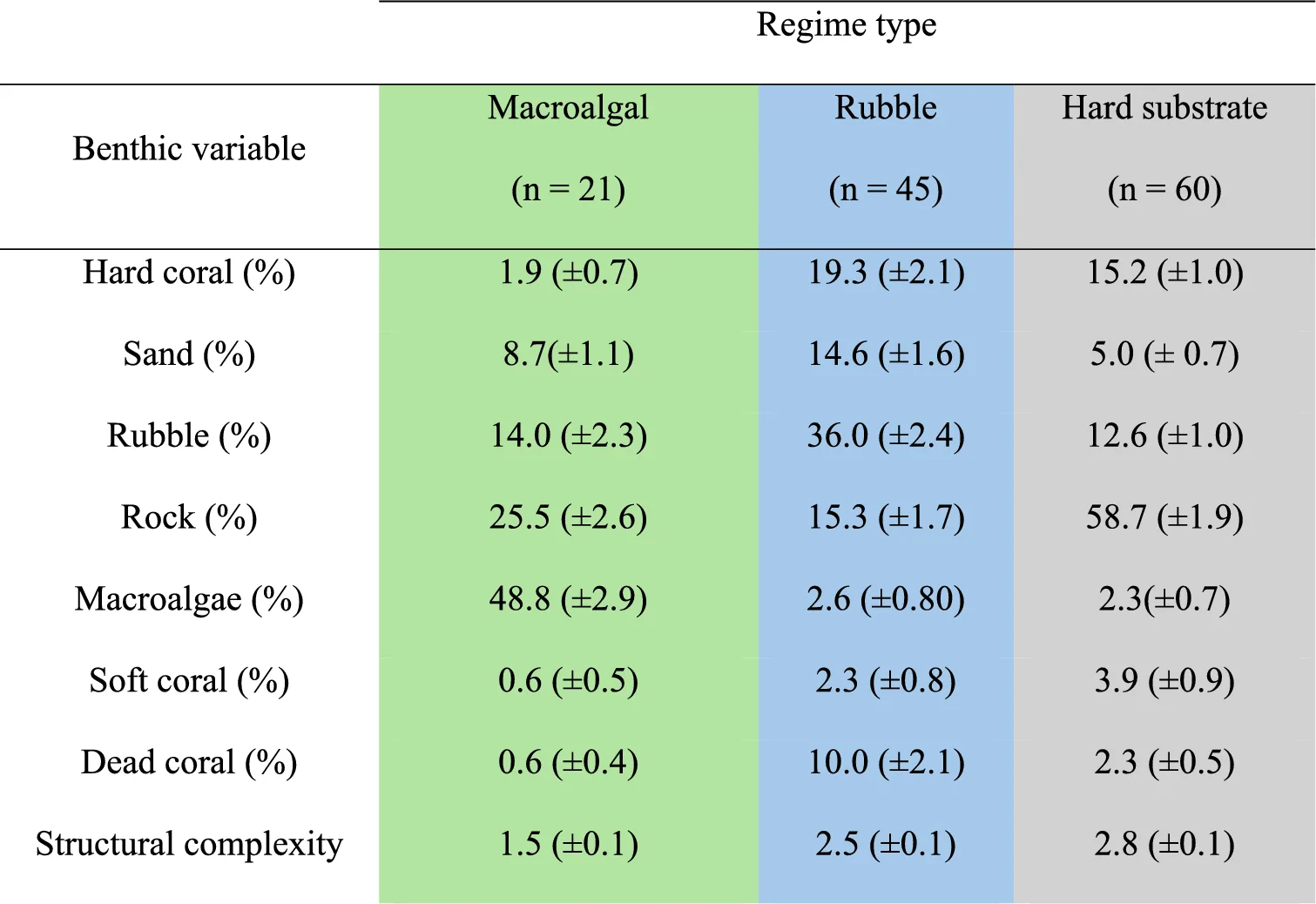
Mean percentage cover (± SE) of each benthic variable and mean structural complexity (± SE) in each benthic regime. The benthic regimes are identified by K-means clustering (Fig. 2). The number of site-year combinations encompassed in each benthic regime is indicated in parentheses. Each site-year combination represents benthic community data averaged over 8–16 point count surveys at a site in a year (1994–2022)

### Differences in invertivorous fish community structure across benthic regimes (1994–2022)

**Fig. 3:**
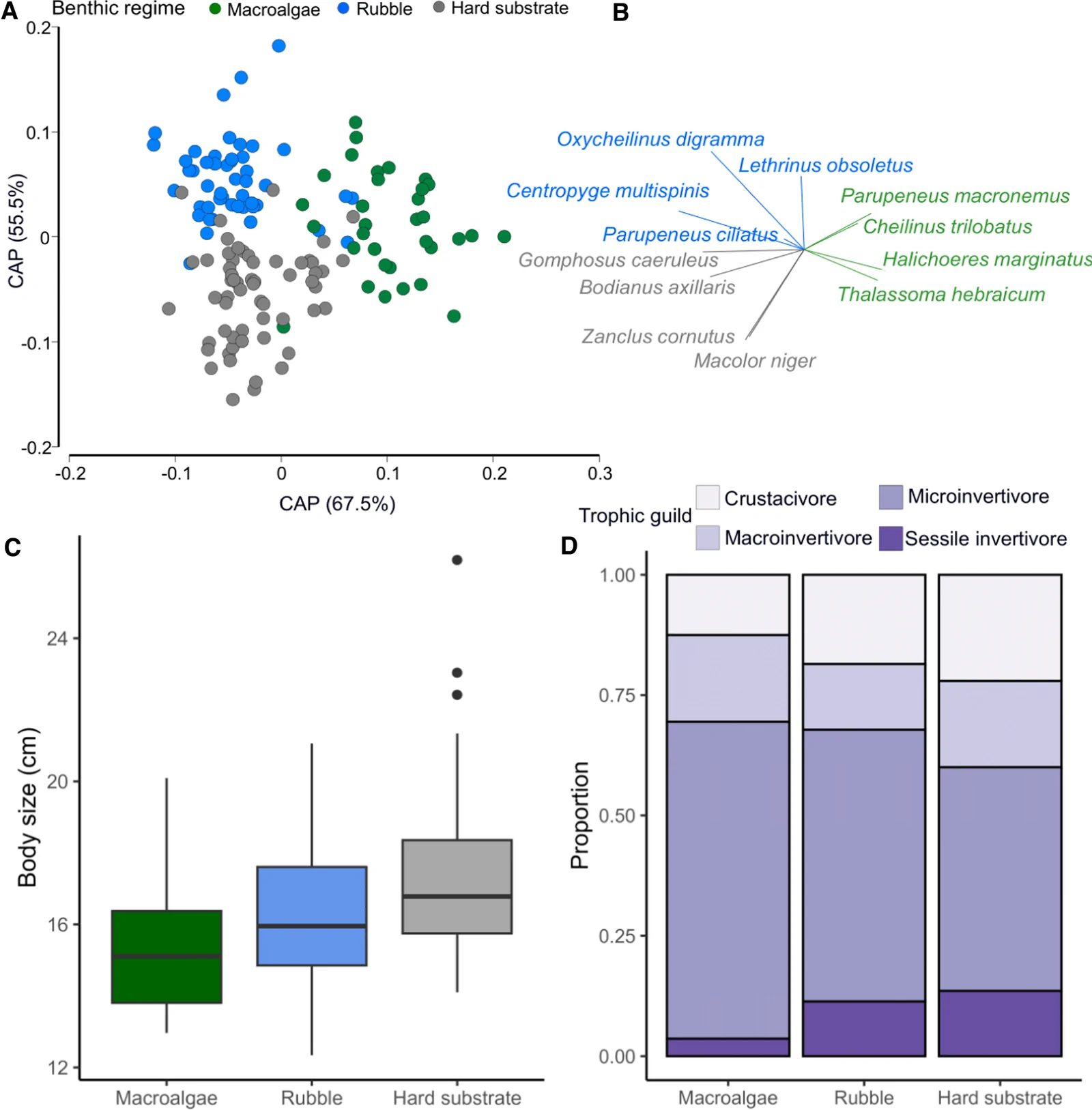
**a** Canonical analysis of principal coordinates (CAP) ordination showing invertivorous fish community patterns across three benthic regimes (squared canonical correlation of each axis shown in parantheses; note these axes are not independent like in a PCA and so sum to more than 100%). Each point represents averaged fish community composition per site per year (1994–2022). **b** Invertivorous reef fish species responsible for driving differences between benthic regimes and colour coded according to its regime-association, determined by its Spearman Rank correlations with the first two canonical ordination axes from the CAP analysis (12 out of 59 species shown for visualisation purposes). The length of each vector line is proportional to the strength of the correlation. **c** Box plots of invertivorous fish body size across three benthic regimes (box: interquartile range with median; whiskers: 1.5 × interquartile range; points are outliers). **d** Abundance of crustacivores, macroinvertivores, microinvertivores and sessile invertivores within each benthic regime, expressed as a proportion of total abundance

There were distinct differences in invertivorous fish community composition among benthic regimes (Fig. 3a,b). Fish communities from the macroalgal, rubble and hard substrate regimes were correctly classified with an allocation success of 95.2%, 84.4% and 81.7%, respectively (Table S2), well above the 33.3% threshold, and therefore indicative of distinct community types than expected by chance alone. Invertivorous fish body size differed significantly among benthic regimes (Pseudo-*F*_2_ = 8.65, *p* < 0.001), with the hard substrate regime harbouring fishes 2.6 cm larger than the macroalgal regime (*t* = 3.64, *p* < 0.001), and 1.6 cm larger fish than those on the rubble regime (*t* = 2.77, *p* < 0.01). The macroalgal and rubble regimes did not differ in mean fish body size (*t* = 1.71, *p* = 0.09) (Fig. 3c, Table S3).

Abundance of fish in different invertivorous trophic guilds varied among benthic regimes. Benthic regime had a significant effect on crustacivore (Pseudo-*F*_2_ = 5.89, *p* < 0.01), microinvertivore (Pseudo-*F*_2_ = 6.0, *p* < 0.01) and sessile invertivore abundance (Pseudo-*F*_2_ = 13.70, *p* < 0.001) but not macroinvertivore abundance (Pseudo-*F*_2_ = 2.63, *p* = 0.07). The proportional abundance of crustacivores was higher in the hard substrate and rubble regimes than in the macroalgal regime, by 10 and 6 percentage points, respectively (hard substrate vs macroalgal: *t* = 3.24, *p* < 0.01, rubble vs macroalgal: *t* = 3.58, *p* < 0.001) (Fig. 3d, Table S3). However, microinvertivores accounted for a 10 percentage point greater share of the community in the rubble regime than in the hard substrate regime (*t* = 3.46, *p* < 0.001), with no significant differences in microinvertivore abundance between hard substrate and macroalgal regimes (*t* = 0.40, *p* = 0.70) or between rubble and macroalgal regimes (*t* = 1.92, *p* = 0.06). Sessile invertivores made up 10 and 8 percentage points more of the community in the hard substrate and rubble regimes, respectively, compared to the macroalgal regime (hard substrate vs macroalgal: *t* = 5.57, *p* < 0.001, rubble vs macroalgal: *t* = 4.61, *p* < 0.001) (Fig. 3d), but there was no significant difference in sessile invertivore abundance between the hard substrate and rubble regimes (*t* = 0.63, *p* = 0.54) (Table S3).

### Quantification of invertivorous fish communities on coral rubble habitats (2022)

For benthic community surveys conducted in 2022, K-means partitional clustering identified three major benthic clusters (Fig S2), that represented the macroalgal (four sites), rubble (five sites) and hard substrate (nine sites) regimes (Table S4, Fig. S3). CAP analysis performed on invertivorous fish community composition in 2022 showed distinct community differences between benthic regimes with a total allocation success of 77.8% (Table S5). Allocation success for each benthic regime scored well above the 33.3% threshold, with fish communities from macroalgal, rubble and hard substrate regimes correctly classified with an allocation success of 80.0%, 75.0% and 77.8%, respectively (Table S5). Twelve invertivorous fish species were associated with the macroalgal regime, 12 species with the rubble regime and 30 with the hard substrate regime (Table S6). In 2022, a total of 55 invertivorous fish species were counted across all benthic regimes, and the most abundant were *Thalassoma hebraicum* (a microinvertivore representing 15% of total abundance)*, Labroides dimidiatus* (a microinvertivore, 12%)*, Halichoeres marginatus* (a microinvertivore, 10%)*, Halichoeres hortulanus* (a macroinvertivore, 10%)*, Cheilinus trilobatus* (a macroinvertivore, 9.8%)*, Scolopsis frenatus* (a microinvertivore, 9.2%). Mean body size (± SE) of invertivorous fish on coral reefs was 17.6 ± 3.3 cm, ranging from 13.6 to 26.2 cm. Microinvertivores were the most abundant trophic guild (49.1%), followed by crustacivores (26.4%), macroinvertivores (16.3%), and sessile invertivores (8.2%).

Across our experimental surveys in all three benthic regimes, we counted a total of 503 invertivorous fish individuals present on coral rubble habitats, with a total of 178 actively feeding in coral rubble habitats. A total of 38 species were identified, of which the most abundant were *Scolopsis frenatus* (Bridled monocle bream, microinvertivore) representing 17% of all individuals, and *Halichoeres hortulanus* (Checkerboard wrasse, macroinvertivore) and *Parupeneus macronemus* (Long-barbel goatfish, a microinvertivore) representing 15% and 10% of all individuals, respectively. Microinvertivores represented the most abundant trophic guild accounting for 57% of all individuals present and 58% of all individuals feeding, followed by macroinvertivores (23% present and 31% feeding), crustacivores (14% present and 6.8% feeding) and sessile invertivores (6% present and 4% feeding). Mean (± SE) body size of invertivorous fishes present on rubble habitats was 17.9 ± 5.5 cm, ranging from 9.4 to 35 cm, while mean (± SE) body size of invertivorous fish feeding in rubble habitats was 17.0 ± 3.3 cm, ranging from 11.9 to 25.8 cm.

### Effect of rubble disturbance on invertivorous fish community structure on coral rubble habitats (2022)

**Fig. 4:**
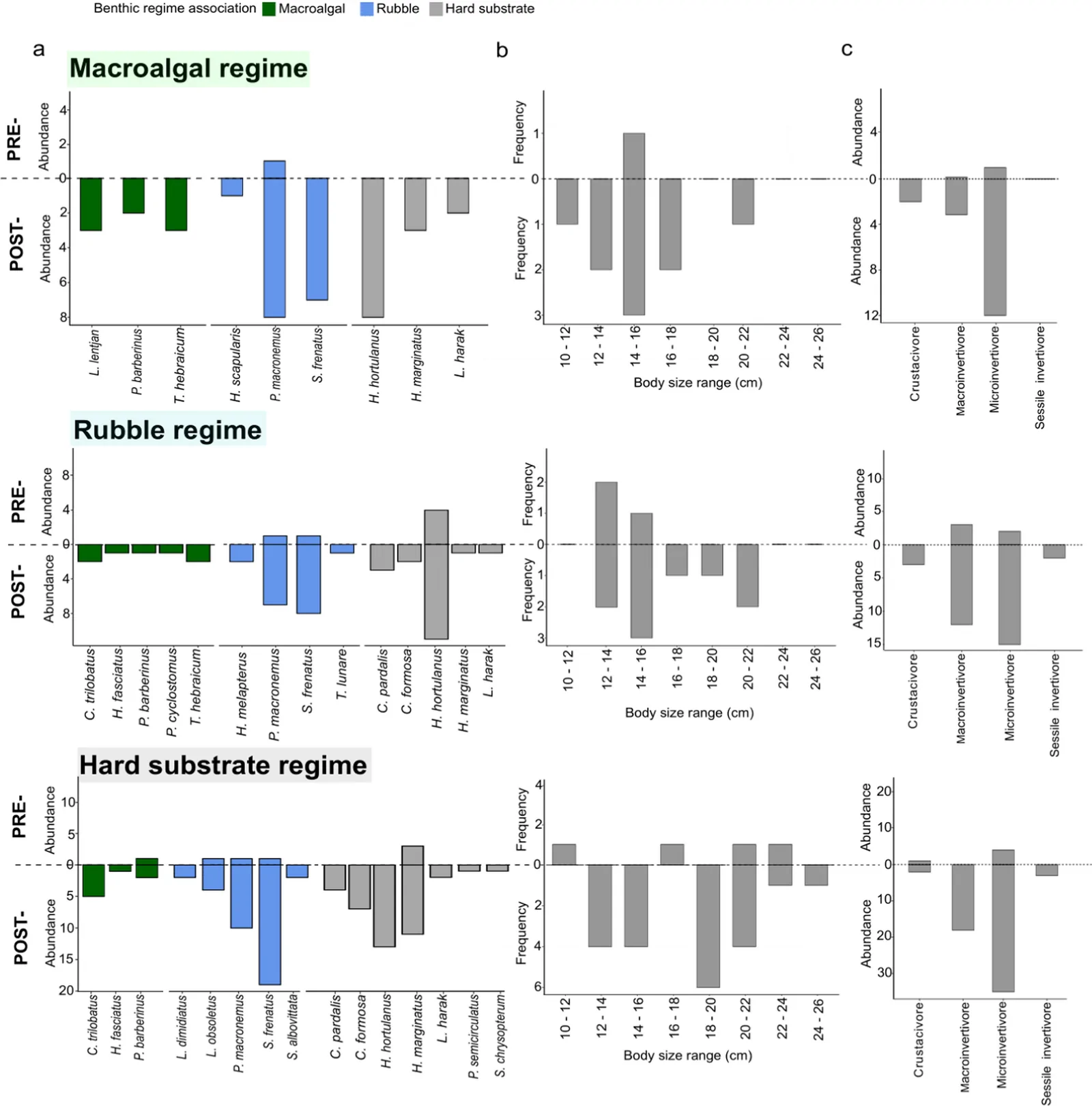
**a** Abundance of invertivorous fish species feeding in coral rubble pre- and post- rubble disturbance. Each species is colour coded according to its regime-association, determined by its Spearman Rank correlations with the first two canonical ordination axes. **b** Frequency of occurrences of body size classes of invertivorous fish observed feeding in coral rubble across benthic regimes, pre- and post-disturbance. **c** Abundance of trophic guilds of invertivorous fish feeding in coral rubble pre- and post- rubble disturbance. Values plotted above zero represent pre-disturbance observations, while values plotted below zero represent post-disturbance observations

Rubble disturbance had a significant effect on invertivorous fish community composition present on rubble habitats (Pseudo-*F*_1_ = 6.75, *p* < 0.01) (Table S7), with average dissimilarity of 77.6% between pre- and post-disturbance community composition. This dissimilarity was driven by a higher abundance of *Scolopsis frenatus, Halichoeres hortulanus,* and *Parupeneus macronemus* post-disturbance, with these species explaining 13.4%, 10.1% and 7.5% of variation in the data, respectively. Rubble disturbance also had a significant effect on the community composition of invertivorous fishes that were recorded feeding in rubble habitats (Pseudo-*F*_1_ = 13.82, *p* < 0.01) (Table S7), with an average dissimilarity of 91.9% in community composition between pre- and post- disturbance. Before rubble disturbance, the principal fishes feeding were *Halichoeres hortulanus* and *Parupeneus macronemus*, contributing 50.4% and 39.5% to within group similarity, respectively (Fig. 4a). After the rubble habitat was disturbed, both species increased in abundance with *Halichoeres hortulanus* and *Parupeneus macronemus,* contributing 20.6% and 11.2% to overall dissimilarity between disturbance levels (Fig. 4a). Additionally, *Scolopsis frenatus* became a more dominant feeder in post-disturbed rubble, contributing 17.4% to dissimilarity. *Parupeneus barberinus* and *Coris formosa*, which were absent in pre-disturbance rubble, also played a moderate role in post-disturbance fish feeding (contributing 6.6% and 5.3% to dissimilarity, respectively).

Although the body size of fishes present and feeding on rubble habitats did not differ before and after rubble disturbance (Pseudo-*F*_1_ = 0.82, *p* = 0.39; Pseudo- *F*_1_ = 3.74, *p* = 0.11), there was a significant effect of rubble disturbance on the variability (standard deviation) of invertivorous fish body sizes present on coral rubble habitats (Pseudo-*F*_1_ = 9.56, *p* < 0.05) and on the variability (standard deviation) of invertivorous fish body sizes feeding in coral rubble habitats (Pseudo-*F*_1_ = 4.33, *p* < 0.05) (Table S7). For both response variables, there was an increase in body size variability after rubble was disturbed (Fig. 4b). The abundances and number of feeding observations for crustacivores (Pseudo-*F*_1_ = 13.9, *p* < 0.01; Pseudo-*F*_1_ = 8.7, *p* < 0.01), macroinvertivores (Pseudo-*F*_1_ = 19.94, *p* < 0.01; Pseudo-*F*_1_ = 13.9, *p* < 0.01), and microinvertivores (Pseudo-*F*_1_ = 21.71, *p* < 0.01; Pseudo-*F*_1_ = 18.36, *p* < 0.01) increased after rubble disturbance (Fig. 4c, Table S7). The number of sessile invertivores present on rubble habitats was also higher following rubble disturbance (Pseudo-*F*_1_ = 8.14, *p* < 0.01), however, the number of sessile invertivores feeding in rubble habitats was not significantly influenced by rubble disturbance (Pseudo-*F*_1_ = 4.26, *p* = 0.05) (Table S7).

### Effect of benthic regime on pre-disturbance and post-disturbance invertivorous fish communities on coral rubble habitats

Community composition of invertivorous fishes observed feeding in pre-disturbance rubble habitats differed significantly among benthic regimes (Pseudo-*F*_2_ = 2.30, *p* < 0.05) (Table S8), due to differences in fish species feeding on the rubble and macroalgal regimes (*t* = 2.1, *p* < 0.05). Community dissimilarity between the rubble and macroalgal regimes was driven by *Halichoeres hortulanus* feeding in the rubble regime but not the macroalgal regime (68.3% of overall dissimilarity between groups), and increased abundance of *Parupeneus macronemus* feeding in the rubble regime (21.5% of overall dissimilarity between groups). There was also a significant effect of benthic regime on mean body size of invertivorous fish present on pre-disturbed rubble habitats (Pseudo-*F*_2_ = 3.81, *p* < 0.05), with larger fish observed on the hard substrate than macroalgal regime (*t* = 2.9, *p* < 0.05). There was no significant effect of benthic regime on the remaining response variables for pre-disturbance (Table S8) or post-disturbance rubble habitats (Table S9).

## Discussion

Coral rubble is becoming an increasingly dominant benthic habitat on tropical coral reefs due to climate-induced coral mortality and subsequent breakdown of coral skeletons (Rogers et al. 2018; Wolfe et al. 2021; Morais et al. 2022). While rubble often represents a degraded and structurally compromised habitat, it also supports a diverse and abundant community of benthic invertebrates, which in turn provide a potential food resource for secondary consumers, particularly invertivorous reef fishes (Kramer et al. 2015, 2016). Yet few studies have quantified the influence of rubble habitats on invertivorous reef fish community structure and how this differs among regime types on coral reefs. Here, we show that invertivorous reef fish community structure is distinct amongst benthic regimes at the reef scale, and that the benthic regime of the reef influences the invertivorous fish community associated with coral rubble habitats on the reef. However, once coral rubble is physically disturbed, differences in invertivorous fish communities among benthic regimes diminish, and a consistent subset of invertivorous reef fish exploit rubble habitats regardless of reef benthic regime. These findings suggest that the naturally dynamic nature of rubble habitats facilitates opportunistic feeding on coral reefs. These findings challenge the perception of coral rubble as a purely degraded habitat and highlight its role in reef trophodynamics.

### Distinct invertivorous reef fish communities across benthic regimes

Invertivorous reef fish community composition was highly distinct among the macroalgal regime, rubble regime and hard substrate regime. This is consistent with studies across the populated Hawaiian Islands where unique combinations of reef fish community structure and varying microhabitat compositions created distinct ecological regimes (Jouffray et al. 2015; Donovan et al. 2018). In the Seychelles, previous studies have typically considered macroalgal or coral dominated regimes when exploring ecological dynamics (Graham et al. 2015). However, our results support previous evidence for multiple benthic regimes in this area (Chong-Seng et al. 2014). While the recovery potential of scleractinian corals across these three benthic regimes has been studied in the Seychelles (Chong-Seng et al. 2014), future research should explore how variation in benthic microhabitat composition within these regimes influences reef fish community structure to better understand the complex ecological dynamics of these reef systems.

The hard substrate regime supported significantly larger-bodied fishes compared to rubble and macroalgal regimes. The prevalence of larger fishes in this regime is likely linked to the presence of granitic reef substrates and live coral cover, which provide structural complexity at a suitable scale for large-bodied fishes (Graham et al. 2006a; Harris et al. 2014). Different invertivorous feeding guilds were associated with specific benthic regimes. Hard substrate and rubble regimes supported higher abundances of crustacivores and sessile invertivores, whilst the rubble regime supported the greatest abundances of microinvertivores. This may reflect prey availability within each benthic regime since microhabitat type is a strong predictor of benthic invertebrate community structure (Enochs and Manzello 2012a; Counsell et al. 2018; Stella et al. 2022). For example, the hard substrate regime (characterised by rock substrate and live coral cover) and the rubble regime, (characterised by coral rubble and live coral cover) both encompass microhabitats that are rich in large crustaceans, like decapods (e.g. Diogenidae, Palaemonidae, Xanthidae, and Galatheidae) and stomatopods (e.g. Gonodactylidae) and provide hard surfaces that facilitate the recruitment of sessile invertebrates (e.g. Porifera and Cnidarians) (Gischler and Ginsburg 1996; Rasser and Riegl 2002; Wolfe et al. 2021; Stella et al. 2022). Additionally, coral rubble hosts a disproportionate abundance of small-bodied crustaceans (e.g. Harpacticoida and Amphipoda) (Stella et al. 2022; Wolfe et al. 2023a). This highlights a potentially important relationship structuring invertivorous reef fish communities. Further studies quantifying benthic invertebrate communities across distinct microhabitats, for example through estimations of invertebrate productivity (Edgar 1990; Fraser et al. 2021), could provide deeper insight into how prey availability influences invertivorous reef fish assemblages on Seychelles inner Islands reefs.

### Invertivorous reef fish community structure on coral rubble habitats

Of all the invertivorous fish species surveyed in 2022, approximately 70% of them were identified in our experimental surveys on coral rubble habitats, but only 38% of them actively fed in coral rubble habitats. This suggests that while most invertivorous fishes traverse coral rubble habitats, only a smaller subset will actively exploit rubble-associated prey, even after physical disturbance reduces search time and makes prey readily accessible. The disparity between presence and feeding activity is consistent with ecological theory, where an animal must balance the benefits of resource acquisition with safety (Brown 2009). Although coral rubble contains abundant invertebrate prey, its low structural complexity provides few refuges for the average-sized invertivorous reef fish. This may limit use of the habitat to species that are more tolerant of risk. In addition to increased predation risk, the small interstitial spaces between rubble pieces may restrict access to prey, meaning that only fishes with morphological adaptations suited to feeding in such confined microhabitats may fully exploit these resources. Furthermore, coral rubble habitats may only provide prey that are energetically profitable or preferred by a subset of invertivorous fishes. Rubble-dwelling invertebrates contribute unevenly to invertivorous reef fish diets, and these fishes exhibit marked dietary specialisation rather than generalised feeding on available prey (Kramer et al. 2015; Kamen et al. 2024). Consequently, readily accessible prey in rubble habitats may not be exploited uniformly across invertivorous fish communities.

The dominant reef fish species identified in our study are from the Labridae, Mullidae and Nemipteridae, families and are often associated with coral rubble habitats ( Kramer et al. 2016; Wolfe et al. 2021). *Halichoeres hortulanus* and *Parupeneus macronemus* were consistently observed feeding in rubble habitats before and after rubble disturbance, suggesting that their morphological and behavioural ecology make them well-suited to exploiting this habitat. Morphologically, *Parupeneus* spp. have hyoid barbels which provide chemosensory cues for detecting prey (Holland 1978), and possess a long, elongated head and snout that may enable them to forage on prey embedded deeper in substrates (Mittelheiser et al. 2022), while *Halichoeres* spp., like other wrasses, comprise powerful pharyngeal jaws which enable them to crush prey (Wainwright 1988). Behaviourally, *Halichoeres* spp. and *Parupeneus* spp. are known to forage in intra- or interspecific groups (Lukoschek and McCormick 2000; Barber and Auster 2005; Jones 2005), a behaviour that may be advantageous in rubble habitats where shelter is limited. *Scolopsis frenatus,* while present in rubble habitats pre-disturbance, only significantly increased its feeding activity after rubble disturbance, suggesting that it may act as an opportunistic attendant species that benefits from substrate disturbance (Boaden and Kingsford 2012).

The mean body size of invertivorous fishes present and feeding in rubble habitats was comparable to that of invertivorous fishes observed in the long-term surveys on coral reefs from 1994 to 2022 in Seychelles. However, the size range of fishes feeding in rubble habitats was narrower than that observed in the long-term data. The absence of large (> 25 cm) and small (< 12 cm) fishes from active feeding observations on rubble indicates that feeding in this habitat is constrained to mid-sized invertivorous fishes. Microinvertivores were the dominant invertivorous feeding guild in both long-term coral reef and coral rubble habitat surveys, reflecting the high availability of their microinvertebrate prey (e.g. copepods, tanaids, and amphipods). These microinvertebrates constitute much of the metazoan biodiversity and abundance on tropical coral reefs, particularly in coral rubble (Monroy-Velázquez et al. 2017; Stella et al. 2022). While coral rubble is projected to sustain trophic pathways in the short to mid-term (Rogers et al. 2018; Morais et al. 2020), its benefits are likely limited to a subset of highly mobile, mid-sized microinvertivores that forage in groups. These species either have morphological adaptations for accessing deep rubble prey or serve as opportunistic attendants, benefiting from the foraging activities of specialised species.

### The effect of physical disturbance and benthic regime on invertivorous reef fish community structure

Coral rubble provides a habitat rich in invertebrate prey, however, invertivorous fishes do not exhibit strong selection for this habitat (Kramer et al. 2016). While habitat structure, prey preferences, and predation risk are known to shape the relationship between invertivorous fishes and their prey (Fulton and Bellwood 2002; Kramer et al. 2016; Chen et al. 2022), our findings suggest that the physically dynamic nature of coral rubble may also influence invertivorous fish use of rubble habitats. After rubble was disturbed, invertivorous fish community structure significantly shifted, consistently increasing the abundance of fishes present or frequency of feeding in rubble habitats. While the disturbance experiment applied here represents a broad assay of disturbance, it reflects the inherently dynamic nature of unconsolidated rubble, where physical processes can intermittently enhance prey accessibility and reduce the energetic costs of searching for and handling prey. These factors facilitate feeding opportunities for invertivorous fishes that would otherwise not use or have reduced use of rubble habitats. Disturbance foraging on coral rubble habitats, that is the act of obtaining food mediated by a physical disturbance, may be facilitated via overturning rubble pieces caused by strong wave surge (Kenyon et al. 2020) or from the feeding activity of fishes, octopuses, or turtles (Sazima et al. 2006, 2007), underscoring the ecological role of unconsolidated rubble on reefs.

Benthic regime influenced the species of invertebrate feeding fishes that target rubble resources. Pre-disturbance composition of fish feeding in rubble habitats within rubble regimes were characterised by *Halichoeres hortulanus* and *Parupeneus macronemus* compared to rubble patches within the macroalgal regime. These patterns likely reflect differences in prey composition and abundance, as algal growth and rubble complexity can shape benthic invertebrate communities (Wolfe et al. 2023b; Goberdhan et al. 2024). Indeed, some fishes will forage for invertebrate fauna that reside on macroalgae, whilst others target rubble and sand surrounding macroalgae (Chen et al. 2022). Mean body size of invertivorous fishes present on coral rubble habitats differed significantly between the hard substrate and macroalgal regimes, a result consistent with our initial finding that the hard substrate regime generally harboured larger-bodied fish, likely due to reef environmental conditions previously outlined that are characteristic of sites in the hard substrate regime (e.g. live coral cover, structural complexity, etc.). These significant differences in community structure on the macroalgal regime compared to the two other regimes, are unsurprising as regime shifts can substantially change associated reef fish communities, altering their community composition, biomass and functional diversity (Graham et al. 2015, 2024). However, after the rubble was disturbed, benthic regime did not have an effect on any aspect of invertivorous reef fish community structure on coral rubble habitats. This suggests that the effects of physical rubble disturbance may mitigate some of the effects of the macroalgal regime on community composition by temporarily resetting habitat conditions and increasing and diversifying the fishes feeding in rubble habitats. This highlights the importance of unconsolidated coral rubble on reefs, which can be manipulated by disturbance to increase prey accessibility for invertivorous fishes; however, the magnitude and frequency of disturbance will likely influence the strength and duration of this resetting effect.

### Implications for future coral rubble studies and coral reef management

Invertivorous reef fish species, like the rockmover wrasse (*Novaculichthys taeniourus*), yellowtailed coris (*Coris gaimard*) and triggerfish species (Balistidae) actively overturn rubble to access buried prey (Randall 1967; Shibuno et al. 1994; Wainwright et al. 2002; Kramer et al. 2016). While these fishes (and similar species of the same genus) have been surveyed on coral reefs in Seychelles (Graham et al. 2007), we observed only one instance of a queen coris (*Coris formosa*) exhibiting rubble-overturning behaviour. It is possible that our three-minute video surveys were insufficient in length to comprehensively document invertivorous fish communities interacting with coral rubble habitats. Additionally, although divers took precautions to minimise disturbances to the natural behaviour of invertivorous reef fishes, the initial setup of the transect line, video cameras and the brief pre-disturbance observation period (3 min) may have inadvertently influenced fish behaviour. This may explain why the dominant species recorded in our surveys (*Parupeneus* spp.*, Halichoeres* spp.), while naturally abundant on coral reefs, are those that tend to be less likely to swim away in the presence of divers (McCormick 1995, Goberdhan, pers. obs.). As such, we would recommend longer survey times in future studies to potentially observe a more comprehensive fish assemblage. Nonetheless, we provide novel and important insights into the foraging ecology of invertivorous reef fish on coral rubble habitats.

Our findings suggest that while few invertivorous reef fish pro-actively feed in coral rubble habitats (Wolfe et al. 2021), disturbance of unconsolidated rubble enhances prey accessibility for species that might otherwise be unable to exploit this resource. Those species that can actively forage in rubble without rubble disturbance, like *Parupeneus macronemus* and *Halichoeres hortulanus*, may have a competitive advantage in future reef environments, and may play a key ecological role in facilitating access to invertebrates in rubble for opportunistic attendant species, potentially influencing trophic interactions and resource use on rubble-dominated reefs.

## Supplementary Information

Below is the link to the electronic supplementary material.

Supplementary file1 (DOCX 631 KB)

## Data Availability

Data is provided within the manuscript or supplementary information files. Raw data are available from the corresponding author upon reasonable request.
