# Supplementary material for: Coral rubble facilitates feeding opportunities for invertivorous reef fishes on tropical coral reefs
Source: Coral Reefs. 2026 Jun 30;45(5):2325–40. doi: 10.1007/s00338-026-02909-9 (PMC13649883; doi:10.1007/s00338-026-02909-9)
Supplement: Supplementary file 1 [file 338_2026_2909_MOESM1_ESM.docx]

**SUPPLEMENTARY MATERIALS**

**Coral rubble facilitates feeding opportunities for invertivorous reef fishes on tropical coral reefs**

Lisa S. Goberdhan^1*^, Gareth J. Williams^1^, Shaun K. Wilson^2,3^, Rodney Melanie^4^, Nicholas A. J. Graham^5^

^1^School of Ocean Sciences, Bangor University, Menai Bridge, Anglesey, LL59 5AB, UK

^2^Australian Institute of Marine Science, Crawley, Western Australia, Australia

^3^Ocean Institute, University of Western Australia, Crawley, Western Australia, Australia

^4^Seychelles Fisheries Authority, Victoria, Mahe, Seychelles

^5^Lancaster Environment Centre, Lancaster University, Lancaster, LA1 4YQ, UK

[*l.goberdhan@bangor.ac.uk](mailto:*l.goberdhan@bangor.ac.uk)

**Table S1.** List of invertivorous reef fish species recorded in this study and their corresponding trophic guild assignments (crustacivores, macroinvertivores, microinvertivores, sessile invertivores), based on global gut content synthesis by Parravicini et al. (2020).

| **Species** | **Trophic guild** |
| --- | --- |
| Cephalopholis leopardus | Crustacivore |
| Cephalopholis urodeta | Crustacivore |
| Cheilinus fasciatus | Crustacivore |
| Epibulus insidiator | Crustacivore |
| Epinephelus coeruleopunctatus | Crustacivore |
| Epinephelus fasciatus | Crustacivore |
| Epinephelus merra | Crustacivore |
| Epinephelus spilotoceps | Crustacivore |
| Lethrinus harak | Crustacivore |
| Lutjanus bohar | Crustacivore |
| Lutjanus fulviflamma | Crustacivore |
| Lutjanus gibbus | Crustacivore |
| Lutjanus kasmira | Crustacivore |
| Lutjanus monostigma | Crustacivore |
| Macolor niger | Crustacivore |
| Oxycheilinus digramma | Crustacivore |
| Parupeneus barberinus | Crustacivore |
| Parupeneus ciliatus | Crustacivore |
| Parupeneus cyclostomus | Crustacivore |
| Parupeneus rubescens | Crustacivore |
| Parupeneus trifasciatus | Crustacivore |
| Bodianus axillaris | Macroinvertivore |
| Cheilinus trilobatus | Macroinvertivore |
| Coris formosa | Macroinvertivore |
| Diagramma pictum | Macroinvertivore |
| Halichoeres hortulanus | Macroinvertivore |
| Lutjanus rivulatus | Macroinvertivore |
| Monotaxis grandoculis | Macroinvertivore |
| Mulloidichthys flavolineatus | Macroinvertivore |
| Novaculichthys taeniourus | Macroinvertivore |
| Plectorhinchus gibbosus | Macroinvertivore |
| Plectorhinchus orientalis | Macroinvertivore |
| Plectorhinchus schotaf | Macroinvertivore |
| Anampses meleagrides | Microinvertivore |
| Chaetodon lunula | Microinvertivore |
| Gomphosus caeruleus | Microinvertivore |
| Halichoeres cosmetus | Microinvertivore |
| Halichoeres marginatus | Microinvertivore |
| Halichoeres scapularis | Microinvertivore |
| Hemigymnus fasciatus | Microinvertivore |
| Hemigymnus melapterus | Microinvertivore |
| Labroides bicolor | Microinvertivore |
| Labroides dimidiatus | Microinvertivore |
| Lethrinus lentjan | Microinvertivore |
| Lethrinus obsoletus | Microinvertivore |
| Macropharyngodon bipartitus | Microinvertivore |
| Parupeneus macronemus | Microinvertivore |
| Scolopsis frenatus | Microinvertivore |
| Stethojulis albovittata | Microinvertivore |
| Sufflamen chrysopterum | Microinvertivore |
| Thalassoma hardwicke | Microinvertivore |
| Thalassoma hebraicum | Microinvertivore |
| Thalassoma lunare | Microinvertivore |
| Apolemichthys trimaculatus | Sessile invertivore |
| Cantherhines pardalis | Sessile invertivore |
| Centropyge multispinis | Sessile invertivore |
| Pomacanthus imperator | Sessile invertivore |
| Pomacanthus semicirculatus | Sessile invertivore |
| Zanclus cornutus | Sessile invertivore |

**Fig. S1.** Elbow method used to identify the optimal number of pre-defined clusters (k). Inflection point inferred at 3 clusters.

**Table S2.** Cross validation results of classifying invertivorous reef fish communities to *a prori* defined benthic regimes.

|  | Classified as | | |  | |
| --- | --- | --- | --- | --- | --- |
| Original group | Hard substrate | Macroalgae | Rubble | Total | Correct Classification (%) |
| Hard substrate | 49 | 4 | 7 | 60 | 81.67 |
| Macroalgae | 1 | 20 | 0 | 21 | 95.24 |
| Rubble | 3 | 4 | 38 | 45 | 84.44 |
| Total correct | 49 | 20 | 38 | 107 | 84.92 |

**Table S3.** Summary statistics of PERMANOVA on variation (a) body size, (b) crustacivore abundance, (c) macroinvertivore abundance (d) microinvertivore abundance (e) sessile invertivore abundance among benthic regimes. Significant values in bold.

|  | Factor | df | SS | Pseudo-F | p |
| --- | --- | --- | --- | --- | --- |
| (a) Body size | Benthic regime | 2 | 77.64 | 8.65 | **0.0003** |
|  | Residuals | 123 | 552.21 |  |  |
|  | Total | 125 |  |  |  |
| (b) Crustacivore | Benthic regime | 2 | 6772.2 | 5.90 | **0.0036** |
|  | Residuals | 123 | 70657 |  |  |
|  | Total | 125 |  |  |  |
| (b) Macroinvertivore | Benthic regime | 2 | 1093.4 | 2.63 | 0.07 |
|  | Residuals | 123 | 25592 |  |  |
|  | Total | 125 |  |  |  |
| (d) Microinvertivore | Benthic regime | 2 | 16069 | 6.00 | **0.0026** |
|  | Residuals | 123 | 1.65E+05 |  |  |
|  | Total | 125 |  |  |  |
| (e) Sessile invertivore | Benthic regime | 2 | 4957.1 | 13.69 | **0.0001** |
|  | Residuals | 123 | 22262 |  |  |
|  | Total | 125 |  |  |  |

**Fig. Fig. S2** PCA diagram with overlaying K-means clustering of the sites. Green dots (n = 4), blue dots (n = 5), and grey dots (n = 9) represent sites color-coded by its respective benthic regime

**Fig. S3** Experimental survey sites (n =17) in 2022 around the inner Seychelles Islands of Mahe, Ste Anne and Praslin. Each coloured dot represents the respective benthic regime of that site in 2022 (blue dots = Rubble regime, green dots = macroalgal regime, grey dots = hard substrate regime)

**Table S4.** Mean benthic cover in each benthic regime expressed as a percentage

| Benthic variable | Macroalgae regime  (n =4) | Rubble regime  (n = 5) | Hard substrate regime  (n = 9) |
| --- | --- | --- | --- |
| Coral (%) | 2.48 | 15.30 | 15.20 |
| Sand (%) | 11.80 | 8.28 | 5.00 |
| Rubble (%) | 11.30 | 53.90 | 14.50 |
| Rock (%) | 33.50 | 15.70 | 59.60 |
| Macroalgae (%) | 40.90 | 0.19 | 3.08 |
| Soft coral (%) | 0.03 | 1.51 | 1.51 |
| Dead coral (%) | 0.00 | 5.75 | 1.10 |

**Table S5.** CAP results showing cross validation results of classifying reef fish communities to benthic regimes

|  | Classified as | | |  | |
| --- | --- | --- | --- | --- | --- |
| Original group | Hard substrate | Macroalgae | Rubble | Total | Correct Classification (%) |
| Hard substrate | 7 | 0 | 2 | 9 | 77.78 |
| Macroalgae | 0 | 4 | 1 | 5 | 80.00 |
| Rubble | 1 | 0 | 3 | 4 | 75.00 |
| Total correct | 7 | 4 | 3 | 14 | 77.78 |

**Table S6.** Species-specific associations of reef fish and benthic regime based on Spearman’s rank correlation coefficient and position along CAP axes for data in the year 2022.

| Species | CAP1 | CAP2 | Benthic regime-association |
| --- | --- | --- | --- |
| Cantherhines pardalis | 0.242954878 | -0.156329566 | Hard substrate |
| Chaetodon lunula | 0.2533742 | -0.308448442 | Hard substrate |
| Cheilinus trilobatus | -0.001323518 | -0.219681297 | Hard substrate |
| Coris formosa | 0.397762646 | 0.029496017 | Hard substrate |
| Epibulus insidiator | 0.669271157 | -0.001028668 | Hard substrate |
| Epinephelus coeruleopunctatus | 0.0424913 | 0.105667631 | Hard substrate |
| Epinephelus merra | 0.163796464 | 0.27473292 | Hard substrate |
| Epinephelus spilotoceps | 0.426439361 | 0.059108075 | Hard substrate |
| Gomphosus caeruleus | 0.448713968 | 0.470361715 | Hard substrate |
| Halichoeres hortulanus | 0.359215304 | -0.321656325 | Hard substrate |
| Halichoeres marginatus | 0.004765591 | -0.21419097 | Hard substrate |
| Hemigymnus melapterus | 0.327667226 | 0.305925195 | Hard substrate |
| Labroides dimidiatus | 0.0395666 | 0.223002062 | Hard substrate |
| Lethrinus harak | 0.094646625 | 0.034495932 | Hard substrate |
| Lutjanus bohar | 0.408380238 | 0.045753887 | Hard substrate |
| Lutjanus fulviflamma | 0.578227571 | -0.416120829 | Hard substrate |
| Lutjanus gibbus | 0.375013378 | -0.281189743 | Hard substrate |
| Lutjanus kasmira | 0.095407371 | -0.177881028 | Hard substrate |
| Macolor niger | 0.308689327 | -0.220566646 | Hard substrate |
| Mulloidichthys flavolineatus | 0.418774133 | -0.253479634 | Hard substrate |
| Oxycheilinus digramma | 0.35725833 | 0.308505645 | Hard substrate |
| Parupeneus ciliatus | 0.056197551 | 0.050769063 | Hard substrate |
| Parupeneus cyclostomus | 0.114122549 | -0.064360634 | Hard substrate |
| Parupeneus trifasciatus | 0.125608225 | 0.092323296 | Hard substrate |
| Plectorhinchus orientalis | 0.133713202 | -0.490342848 | Hard substrate |
| Plectorhinchus schotaf | 0.241013627 | -0.293532244 | Hard substrate |
| Pomacanthus imperator | 0.411364577 | -0.146912497 | Hard substrate |
| Pomacanthus semicirculatus | 0.189583285 | -0.441390349 | Hard substrate |
| Sufflamen chrysopterum | 0.219639652 | -0.069221691 | Hard substrate |
| Zanclus cornutus | 0.474676395 | 0.024703479 | Hard substrate |
| Diagramma pictum | -0.271910118 | -0.031226576 | Macroalgae |
| Hemigymnus fasciatus | -0.036156546 | 0.124745306 | Macroalgae |
| Lethrinus lentjan | -0.191047393 | -0.16730989 | Macroalgae |
| Lethrinus obsoletus | -0.126511758 | 0.216980911 | Macroalgae |
| Macropharyngodon bipartitus | -0.32161731 | 0.038405375 | Macroalgae |
| Monotaxis grandoculis | -0.040049454 | -0.14593678 | Macroalgae |
| Novaculichthys taeniourus | -0.185681616 | 0.21096026 | Macroalgae |
| Parupeneus barberinus | -0.254327352 | 0.08430446 | Macroalgae |
| Parupeneus macronemus | -0.474675137 | 0.328938402 | Macroalgae |
| Parupeneus rubescens | -0.360754452 | 0.04985229 | Macroalgae |
| Scolopsis frenatus | -0.105965133 | 0.203587203 | Macroalgae |
| Stethojulis albovittata | -0.443095569 | 0.323840795 | Macroalgae |
| Thalassoma hebraicum | -0.123768591 | -0.441220605 | Macroalgae |
| Lutjanus monostigma | 0 | 0 | NA |
| Lutjanus rivulatus | 0 | 0 | NA |
| Plectorhinchus gibbosus | 0 | 0 | NA |
| Thalassoma hardwicke | 0 | 0 | NA |
| Anampses meleagrides | 0.019654273 | 0.446158364 | Rubble |
| Apolemichthys trimaculatus | -0.092800733 | 0.282376327 | Rubble |
| Bodianus axillaris | 0.335122099 | 0.550238852 | Rubble |
| Centropyge multispinis | 0.066333603 | 0.671980244 | Rubble |
| Cephalopholis leopardus | -0.019690727 | 0.433002649 | Rubble |
| Cephalopholis urodeta | 0.131028416 | 0.438865609 | Rubble |
| Cheilinus fasciatus | -0.001328242 | 0.204267256 | Rubble |
| Epinephelus fasciatus | -0.197570116 | 0.301700142 | Rubble |
| Halichoeres cosmetus | -0.001328242 | 0.204267256 | Rubble |
| Halichoeres scapularis | -0.09620582 | 0.619212248 | Rubble |
| Labroides bicolor | 0.203126203 | 0.581383062 | Rubble |
| Thalassoma lunare | -0.107374742 | 0.332201887 | Rubble |

**Table S7.** Summary statistics of permutational analysis of variance (PERMANOVA) testing the effect of rubble disturbance on site-level response variables (n = 17) either present on coral rubble habitats or actively feeding in coral rubble habitats - (a) invertivorous fish community composition present (b) invertivorous fish community composition feeding (c) mean body size of invertivorous fish present (d) mean body size of invertivorous fish feeding (e) standard deviation of invertivorous fish present (f) standard deviation of invertivorous fish feeding (g) crustacivore abundance present (h) crustacivore abundance feeding (i) macroinvertivore abundance present (j) macroinvertivore abundance feeding (k) microinvertivore abundance present (l) microinvertivore abundance feeding (m) sessile invertivore abundance presence (n) sessile invertivore abundance feeding

|  | Factor | df | SS | Pseudo-F | p |
| --- | --- | --- | --- | --- | --- |
| (a) Community composition present | Disturbance | 1 | 4210 | 6.75 | **p < 0.01** |
|  | Location | 16 | 44097 | 4.42 | **p < 0.01** |
|  | Residuals | 16 | 14379.00 |  |  |
|  |  |  |  |  |  |
| (b) Community composition feeding | Disturbance | 1 | 7596.1 | 13.824 | **p < 0.01** |
|  | Location | 16 | 13345 | 1.5179 | **p < 0.05** |
|  | Residuals | 16 | 8792 |  |  |
|  |  |  |  |  |  |
| (c) Mean body size present | Disturbance | 1 | 5704 | 0.82 | 0.39 |
|  | Location | 16 | 191.38 | 2.7368 | **p < 0.01** |
|  | Residuals | 16 | 69.929 |  |  |
|  |  |  |  |  |  |
| (d) Mean body size feeding | Disturbance | 1 | 4.9155 | 3.7388 | p = 0.11 |
|  | Location | 16 | 158.98 | 7.5578 | **p < 0.05** |
|  | Residuals | 16 | 623.65 |  |  |
|  |  |  |  |  |  |
| (e) SD body size present | Disturbance | 1 | 23.94 | 9.5581 | **p < 0.01** |
|  | Location | 16 | 65.101 | 1.6245 | 0.17 |
|  | Residuals | 16 | 40.076 |  |  |
|  |  |  |  |  |  |
| (f) SD body size feeding | Disturbance | 1 | 72.322 | 4.325 | **p < 0.05** |
|  | Location | 16 | 615.75 | 2.3014 | 0.17 |
|  | Residuals | 16 | 83.61 |  |  |
|  |  |  |  |  |  |
| (g) Crustacivore abundance present | Disturbance | 1 | 3.2426 | 13.93 | **p < 0.01** |
|  | Location | 16 | 32.88 | 10.974 | **p < 0.05** |
|  | Residuals | 16 | 21.12 | 5.2157 |  |
|  |  |  |  |  |  |
| (h) Crustacivore abundance feeding | Disturbance | 1 | 0.11765 | 8.7273 | **p < 0.01** |
|  | Location | 16 | 0.34641 | 1.6061 | 0.1824 |
|  | Residuals | 16 | 1.94 |  |  |
|  |  |  |  |  |  |
| (i) Macroinvertivore abundance present | Disturbance | 1 | 2.844 | 19.948 | **p < 0.01** |
|  | Location | 16 | 0.86642 | 6.0774 | **p < 0.01** |
|  | Residuals | 16 | 2.281 |  |  |
|  |  |  |  |  |  |
| (j) Macroinvertivore abundance feeding | Disturbance | 1 | 2.9412 | 13.873 | **p < 0.01** |
|  | Location | 16 | 4.7647 | 1.4046 | 0.2205 |
|  | Residuals | 16 | 3.3922 |  |  |
|  |  |  |  |  |  |
| (k) Microinvertivore abundance present | Disturbance | 1 | 15.559 | 21.706 | **p < 0.01** |
|  | Location | 16 | 29.083 | 2.5358 | **p < 0.01** |
|  | Residuals | 16 | 11.469 |  |  |
|  |  |  |  |  |  |
| (l) Microinvertivore abundance feeding | Disturbance | 1 | 9.8856 | 18.36 | **p < 0.01** |
|  | Location | 16 | 9.8301 | 1.14 | 0.39 |
|  | Residuals | 16 | 8.6144 |  |  |
|  |  |  |  |  |  |
| (m) Sessile invertivore abundance present | Disturbance | 1 | 0.51062 | 8.1433 | **p < 0.01** |
|  | Location | 16 | 2.0817 | 2.0749 | 0.08 |
|  | Residuals | 16 | 1.0033 |  |  |
|  |  |  |  |  |  |
| (n) Sessile invertivore abundance feeding | Disturbance | 1 | 0.081699 | 4.2553 | 0.06 |
|  | Location | 16 | 0.30719 | 1.00 | 0.43 |
|  | Residuals | 16 | 0.30719 |  |  |

**Table S8.** Summary statistics of PERMANOVA testing the effect of benthic regime on pre-disturbance site-level response variables either present on coral rubble habitats or actively feeding in coral rubble habitats - (a) invertivorous fish community composition present (b) invertivorous fish community composition feeding (c) mean body size of invertivorous fish present (d) mean body size of invertivorous fish feeding (e) standard deviation of invertivorous fish present (f) standard deviation of invertivorous fish feeding (g) crustacivore abundance present (h) crustacivore abundance feeding (i) macroinvertivore abundance present (j) macroinvertivore abundance feeding (k) microinvertivore abundance present (l) microinvertivore abundance feeding (m) sessile invertivore abundance presence (n) sessile invertivore abundance feeding

|  | Factor | df | SS | Pseudo-F | p |
| --- | --- | --- | --- | --- | --- |
| (a) Community composition present | Benthic regime | 2 | 2302.7 | 0.69154 | 0.7967 |
|  | Residuals | 14 | 38986.00 |  |  |
|  |  |  |  |  |  |
| (b) Community composition feeding | Benthic regime | 2 | 1503.5 | 2.2848 | **p = 0.0498** |
|  | Residuals | 14 | 4606.2 |  |  |
|  |  |  |  |  |  |
| (c) Mean body size present | Benthic regime | 2 | 62.425 | 3.8079 | **p < 0.05** |
|  | Residuals | 14 | 114.76 |  |  |
|  |  |  |  |  |  |
| (d) Mean body size feeding | Benthic regime | 2 | 16.252 | 2.7791 | 0.3 |
|  | Residuals | 14 | 8.7719 |  |  |
|  |  |  |  |  |  |
| (e) SD body size present | Benthic regime | 2 | 1.1018 | 0.12028 | 0.884 |
|  | Residuals | 14 | 64.123 |  |  |
|  |  |  |  |  |  |
| (f) SD body size feeding | Benthic regime | 2 | 0.95579 | 0.15355 | 1 |
|  | Residuals | 14 | 9.3368 |  |  |
|  |  |  |  |  |  |
| (g) Crustacivore abundance present | Benthic regime | 2 | 1.65 | 0.13835 | 0.8886 |
|  | Residuals | 14 | 2.0958 |  |  |
|  |  |  |  |  |  |
| (h) Crustacivore abundance feeding | Benthic regime | 2 | 0.0073529 | 0.52941 | 1.00 |
|  | Residuals | 14 |  |  |  |
|  |  |  |  |  |  |
| (i) Macroinvertivore abundance present | Benthic regime | 2 | 0.17059 | 0.25169 | 0.8173 |
|  | Residuals | 14 | 4.7444 |  |  |
|  |  |  |  |  |  |
| (j) Macroinvertivore abundance feeding | Benthic regime | 2 | 0.19118 | 16.059 | **p < 0.01** |
|  | Residuals | 14 | 0.083333 |  |  |
|  |  |  |  |  |  |
| (k) Microinvertivore abundance present | Benthic regime | 2 | 2.7081 | 1.3719 | 0.2844 |
|  | Residuals | 14 | 13.818 |  |  |
|  |  |  |  |  |  |
| (l) Microinvertivore abundance feeding | Benthic regime | 2 | 0.035294 | 0.18843 | 0.88 |
|  | Residuals | 14 | 1.3111 |  |  |
|  |  |  |  |  |  |
| (m) Sessile invertivore abundance present | Benthic regime | 2 | 0.17549 | 2.799 | 0.0979 |
|  | Residuals | 14 |  |  |  |
|  |  |  |  |  |  |
| (n) Sessile invertivore abundance feeding | Benthic regime | 2 | 0.00 | Denominator is 0 |  |
|  | Residuals | 14 | 0.00 |  |  |

**Table S9.** Summary statistics of PERMANOVA testing the effect of benthic on post-disturbance site-level response variables either present on coral rubble habitats or actively feeding in coral rubble habitats - (a) invertivorous fish community composition present (b) invertivorous fish community composition feeding (c) mean body size of invertivorous fish present (d) mean body size of invertivorous fish feeding (e) standard deviation of invertivorous fish present (f) standard deviation of invertivorous fish feeding (g) crustacivore abundance present (h) crustacivore abundance feeding (i) macroinvertivore abundance present (j) macroinvertivore abundance feeding (k) microinvertivore abundance present (l) microinvertivore abundance feeding (m) sessile invertivore abundance presence (n) sessile invertivore abundance feeding

|  | Factor | df | SS | Pseudo-F | p |
| --- | --- | --- | --- | --- | --- |
| (a) Community composition present | Benthic regime | 2 | 3096.7 | 0.85465 | 0.6602 |
|  | Residuals | 14 | 25364 |  |  |
|  |  |  |  |  |  |
| (b) Community composition feeding | Benthic regime | 2 | 2004.2 | 0.82123 | 0.6151 |
|  | Residuals | 14 | 17083 |  |  |
|  |  |  |  |  |  |
| (c) Mean body size present | Benthic regime | 2 | 5.4893 | 0.48863 | 0.6163 |
|  | Residuals | 14 | 78.638 |  |  |
|  |  |  |  |  |  |
| (d) Mean body size feeding | Benthic regime | 2 | 13.752 | 0.75927 | 0.487 |
|  | Residuals | 14 | 126.78 |  |  |
|  |  |  |  |  |  |
| (e) SD body size present | Benthic regime | 2 | 2.9386 | 0.55574 | 0.5812 |
|  | Residuals | 14 | 37.014 |  |  |
|  |  |  |  |  |  |
| (f) SD body size feeding | Benthic regime | 2 | 1.8564 | 0.12523 | 0.8886 |
|  | Residuals | 14 | 148.24 |  |  |
|  |  |  |  |  |  |
| (g) Crustacivore abundance present | Benthic regime | 2 | 1.2967 | 0.71162 | 0.5641 |
|  | Residuals | 14 | 12.756 |  |  |
|  |  |  |  |  |  |
| (h) Crustacivore abundance feeding | Benthic regime | 2 | 0.074183 | 1.3546 | 0.3742 |
|  | Residuals | 14 | 0.38333 |  |  |
|  |  |  |  |  |  |
| (i) Macroinvertivore abundance present | Benthic regime | 2 | 0.73709 | 0.49178 | 0.6224 |
|  | Residuals | 14 | 10.492 |  |  |
|  |  |  |  |  |  |
| (j) Macroinvertivore abundance feeding | Benthic regime | 2 | 1.5824 | 1.76 | 0.21 |
|  | Residuals | 14 | 6.3 |  |  |
|  |  |  |  |  |  |
| (k) Microinvertivore abundance present | Benthic regime | 2 | 0.37892 | 0.11217 | 0.9003 |
|  | Residuals | 14 | 23.647 |  |  |
|  |  |  |  |  |  |
| (l) Microinvertivore abundance feeding | Benthic regime | 2 | 1.3397 | 0.59511 | 0.5897 |
|  | Residuals | 14 | 15.758 |  |  |
|  |  |  |  |  |  |
| (m) Sessile invertivore abundance present | Benthic regime | 2 | 0.16364 | 0.49655 | 0.6103 |
|  | Residuals | 14 |  |  |  |
|  |  |  |  |  |  |
| (n) Sessile invertivore abundance feeding | Benthic regime | 2 | 0.072712 | 0.93967 | 0.56 |
|  | Residuals | 14 | 0.54167 |  |  |
